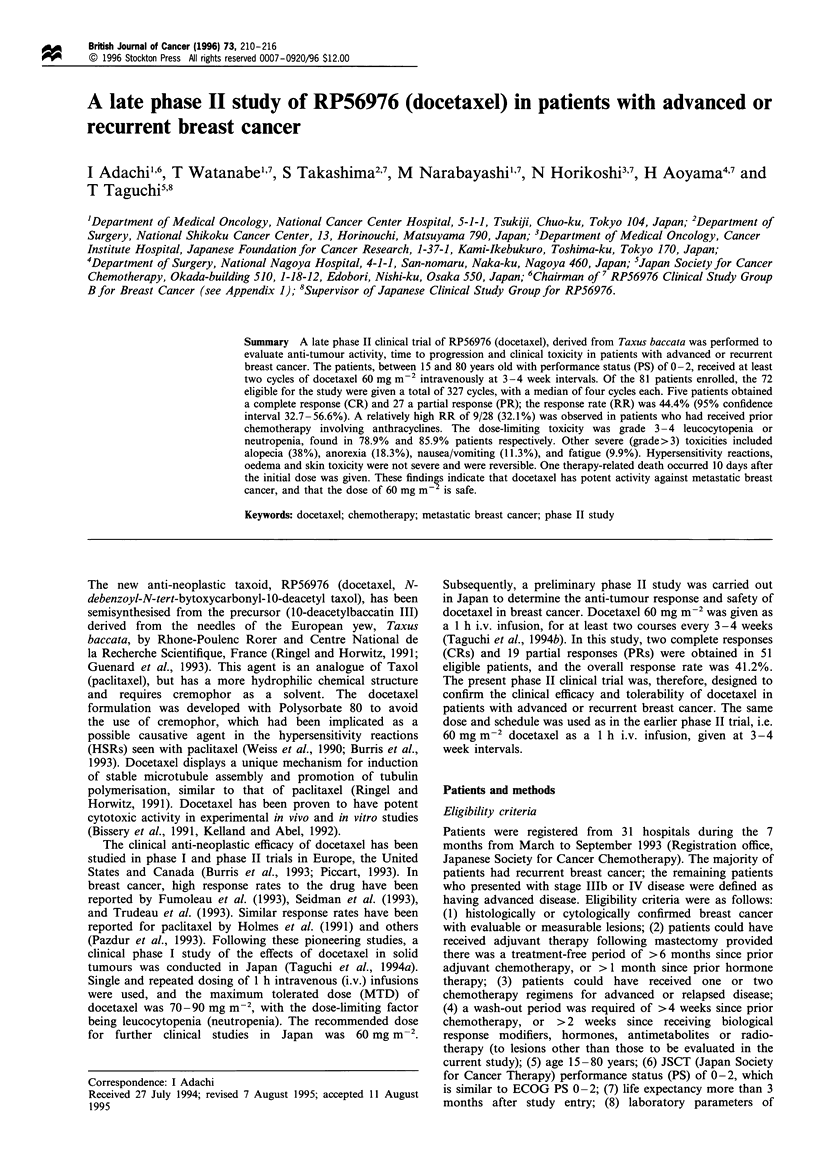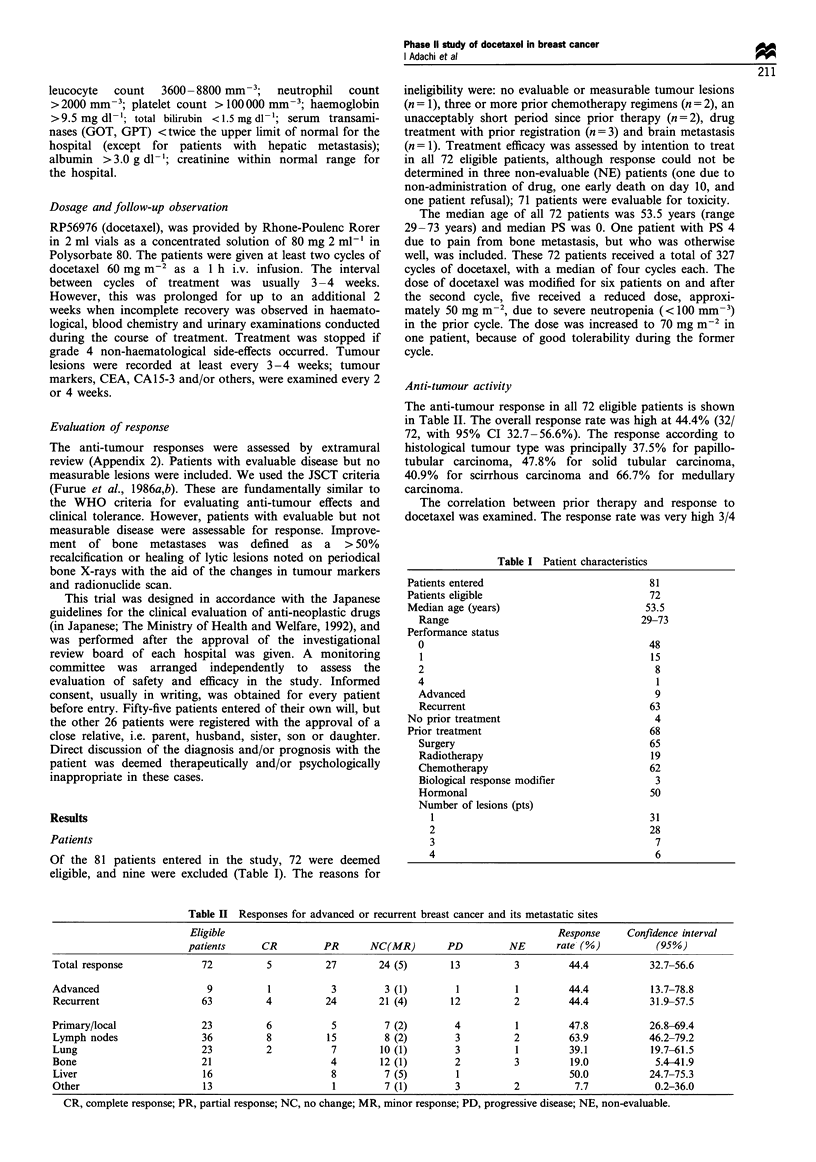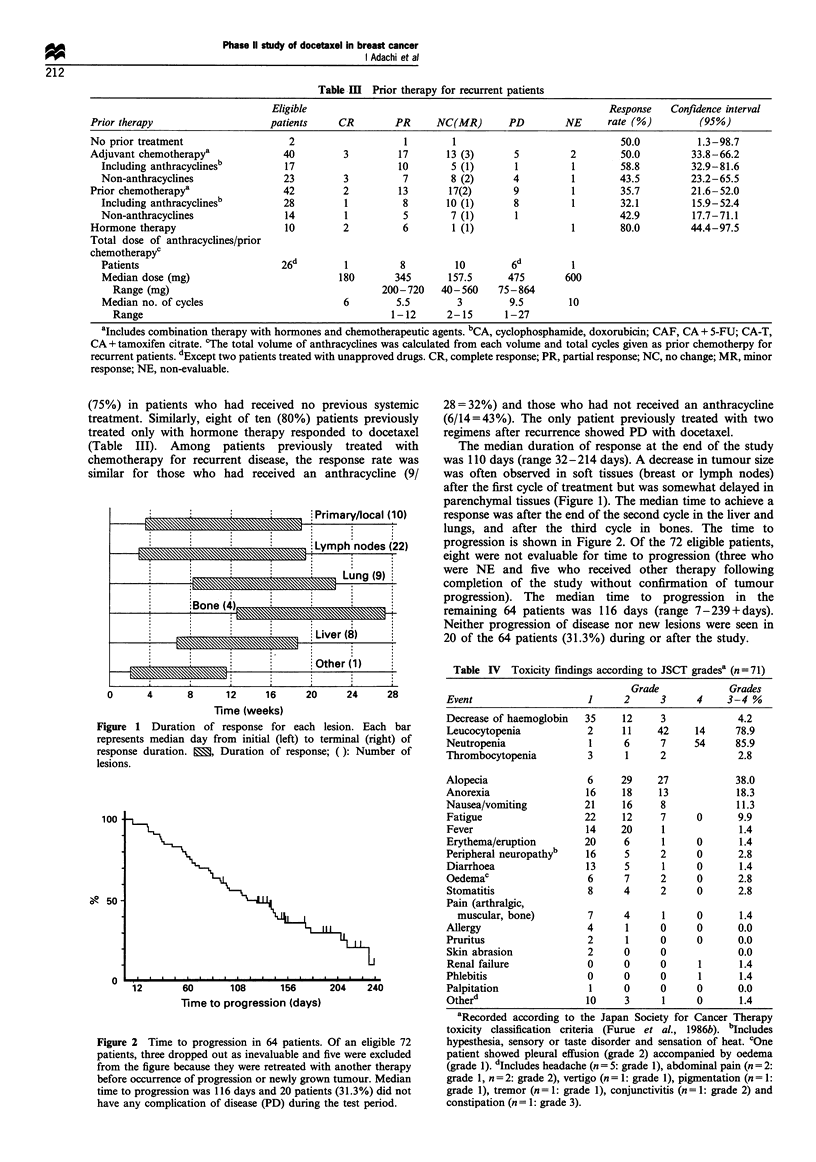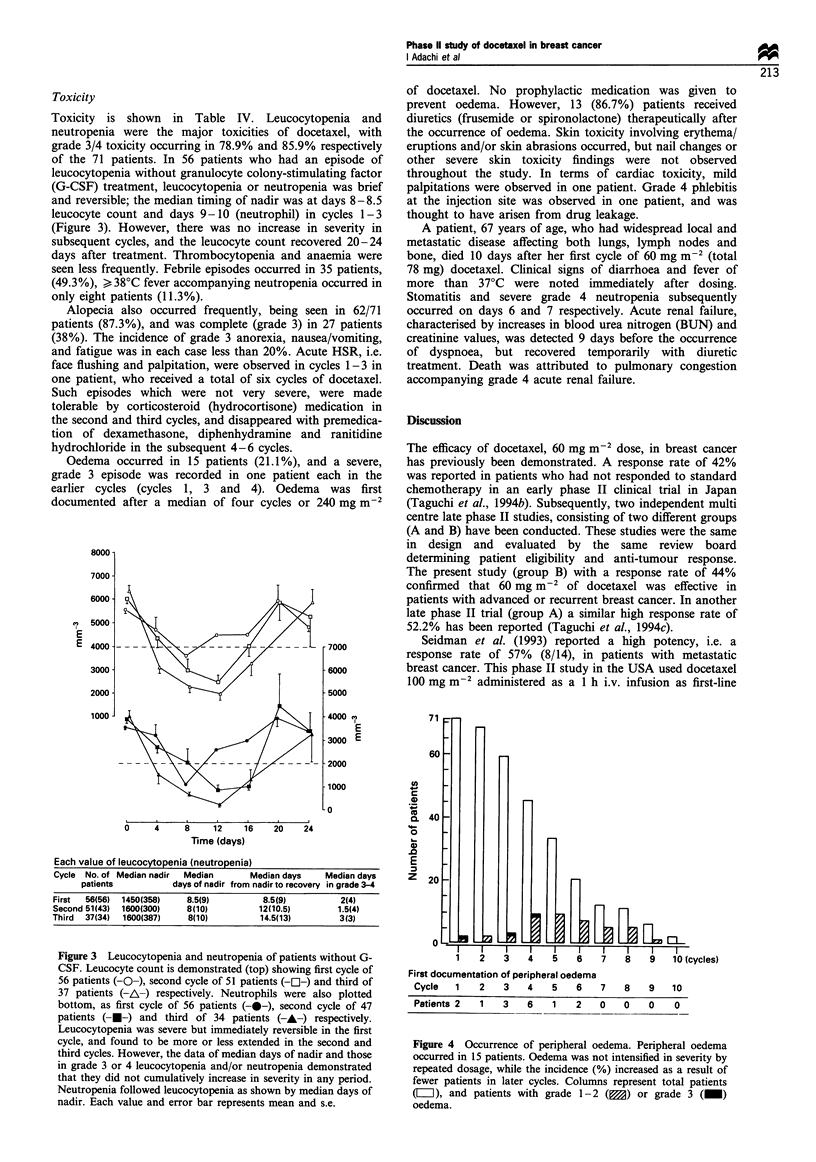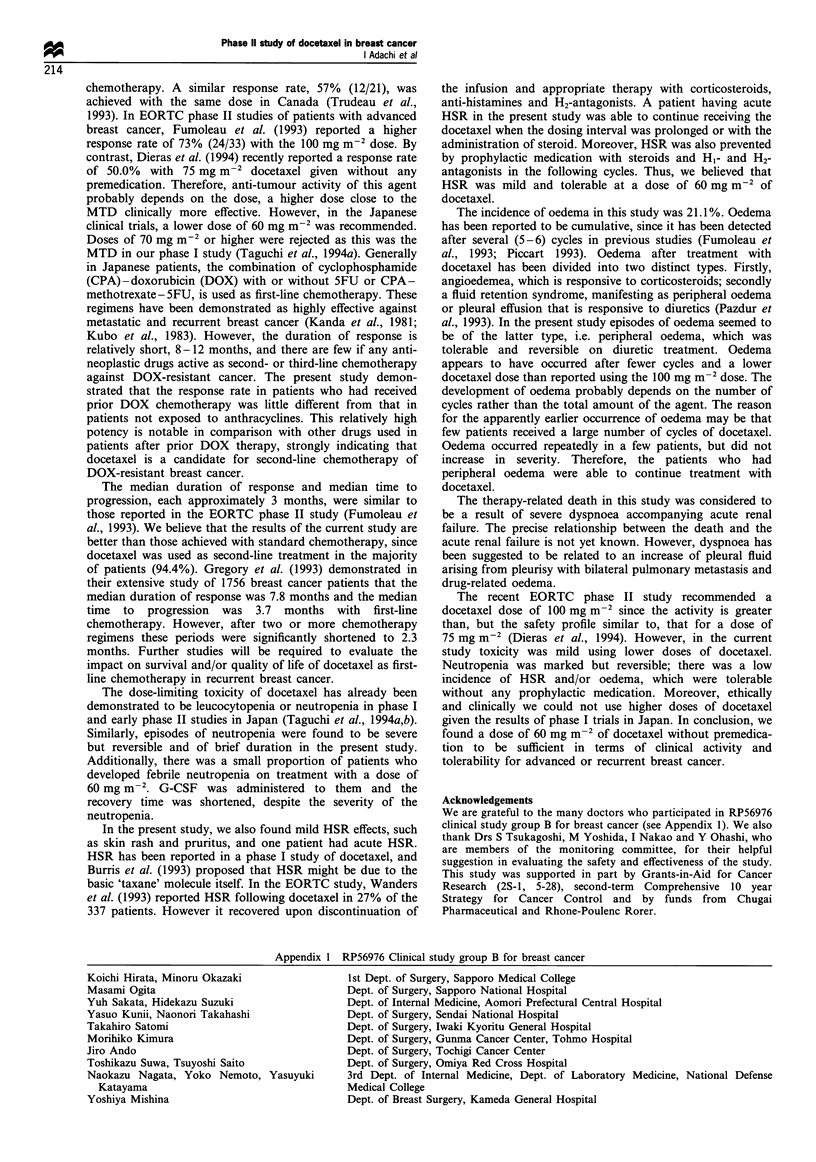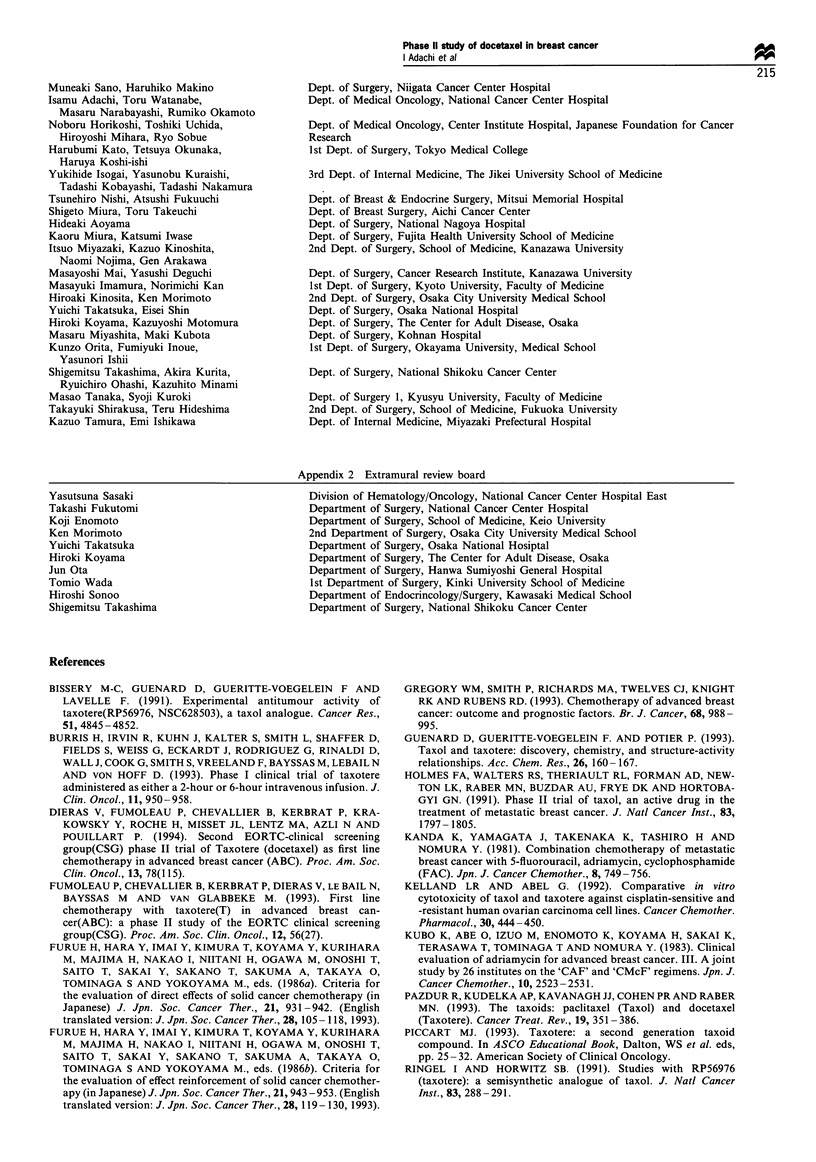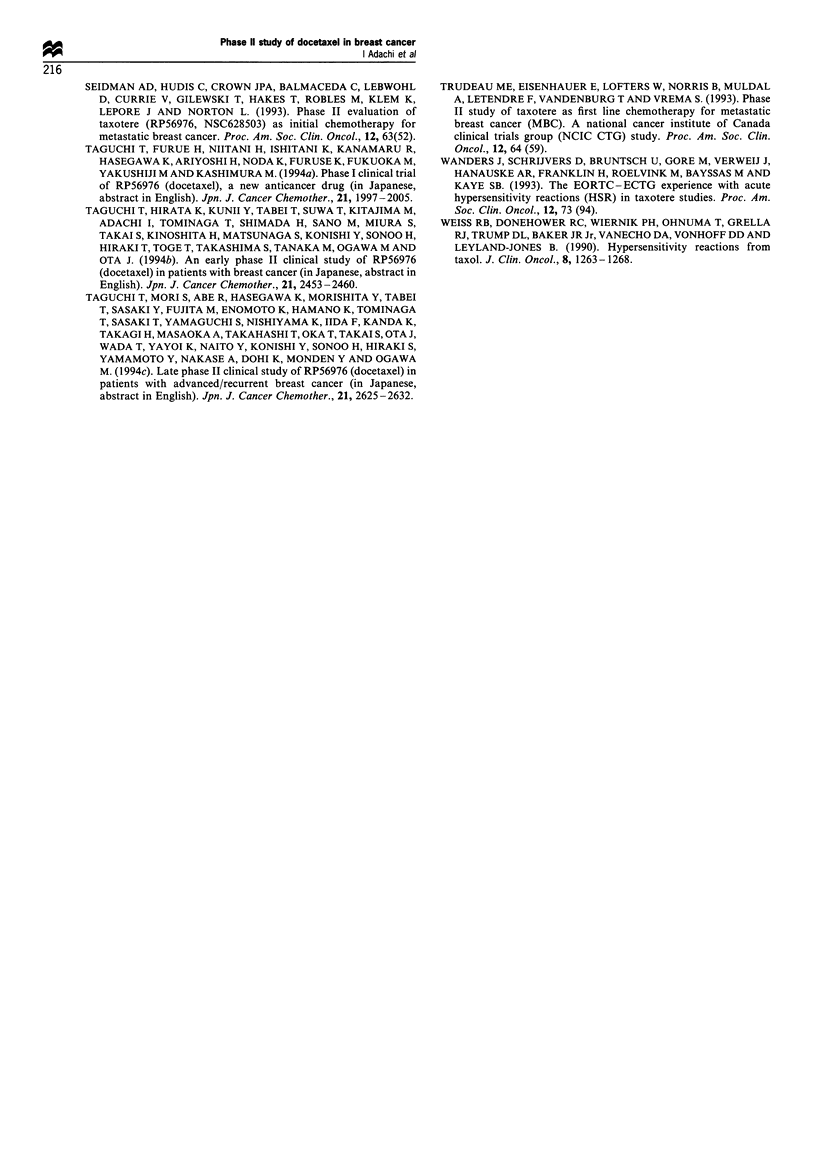# A late phase II study of RP56976 (docetaxel) in patients with advanced or recurrent breast cancer.

**DOI:** 10.1038/bjc.1996.37

**Published:** 1996-01

**Authors:** I. Adachi, T. Watanabe, S. Takashima, M. Narabayashi, N. Horikoshi, H. Aoyama, T. Taguchi

**Affiliations:** Department of Medical Oncology, National Cancer Center Hospital, Tokyo, Japan.

## Abstract

A late phase II clinical trial of RP56976 (docetaxel), derived from Taxus baccata was performed to evaluate anti-tumour activity, time to progression and clinical toxicity in patients with advanced or recurrent breast cancer. The patients, between 15 and 80 years old with performance status (PS) of 0-2, received at least two cycles of docetaxel 60 mg m-2 intravenously at 3-4 week intervals. Of the 81 patients enrolled, the 72 eligible for the study were given a total of 327 cycles, with a median of four cycles each. Five patients obtained a complete response (CR) and 27 a partial response (PR); the response rate (RR) was 44.4% (95% confidence interval 32.7-56.6%). A relatively high RR of 9/28 (32.1%) was observed in patients who had received prior chemotherapy involving anthracyclines. The dose-limiting toxicity was grade 3-4 leucocytopenia or neutropenia, found in 78.9% and 85.9% patients respectively. Other severe (grade > 3) toxicities included alopecia (38%), anorexia (18.3%), nausea/vomiting (11.3%), and fatigue (9.9%). Hypersensitivity reactions, oedema and skin toxicity were not severe and were reversible. One therapy-related death occurred 10 days after the initial dose was given. These findings indicate that docetaxel has potent activity against metastatic breast cancer, and that the dose of 60 mg m-2 is safe.


British Journal of Cancer (1996) 73, 210-216
© 1996 Stockton Press All rights reserved 0007-0920/96 $12.00

### A late phase II study of RP56976 (docetaxel) in patients with advanced or recurrent breast cancer

I Adachi[1,6], T Watanabe[1,7], S Takashima[2,7], M Narabayashi[1,7], N Horikoshi[3,7], H Aoyama[4,7] and T Taguchi[5,8]

[1]*Department of Medical Oncology, National Cancer Center Hospital, 5-1-1, Tsukiji, Chuo-ku, Tokyo 104, Japan;* [2]*Department of Surgery, National Shikoku Cancer Center, 13, Horinouchi, Matsuyama 790, Japan;* [3]*Department of Medical Oncology, Cancer Institute Hospital, Japanese Foundation for Cancer Research, 1-37-1, Kami-Ikebukuro, Toshima-ku, Tokyo 170, Japan;* [4]*Department of Surgery, National Nagoya Hospital, 4-1-1, San-nomaru, Naka-ku, Nagoya 460, Japan;* [5]*Japan Society for Cancer Chemotherapy, Okada-building 510, 1-18-12, Edobori, Nishi-ku, Osaka 550, Japan;* [6]*Chairman of* [7] *RP56976 Clinical Study Group B for Breast Cancer (see Appendix 1);* [8]*Supervisor of Japanese Clinical Study Group for RP56976.*

**Summary** A late phase II clinical trial of RP56976 (docetaxel), derived from *Taxus baccata* was performed to evaluate anti-tumour activity, time to progression and clinical toxicity in patients with advanced or recurrent breast cancer. The patients, between 15 and 80 years old with performance status (PS) of 0-2, received at least two cycles of docetaxel 60 mg $m^{-2}$ intravenously at 3-4 week intervals. Of the 81 patients enrolled, the 72 eligible for the study were given a total of 327 cycles, with a median of four cycles each. Five patients obtained a complete response (CR) and 27 a partial response (PR); the response rate (RR) was 44.4% (95% confidence interval 32.7-56.6%). A relatively high RR of 9/28 (32.1%) was observed in patients who had received prior chemotherapy involving anthracyclines. The dose-limiting toxicity was grade 3-4 leucocytopenia or neutropenia, found in 78.9% and 85.9% patients respectively. Other severe (grade>3) toxicities included alopecia (38%), anorexia (18.3%), nausea/vomiting (11.3%), and fatigue (9.9%). Hypersensitivity reactions, oedema and skin toxicity were not severe and were reversible. One therapy-related death occurred 10 days after the initial dose was given. These findings indicate that docetaxel has potent activity against metastatic breast cancer, and that the dose of 60 mg $m^{-2}$ is safe.

**Keywords:** docetaxel; chemotherapy; metastatic breast cancer; phase II study

The new anti-neoplastic taxoid, RP56976 (docetaxel, *N-debenzoyl-N-tert*-bytoxycarbonyl-10-deacetyl taxol), has been semisynthesised from the precursor (10-deacetylbaccatin III) derived from the needles of the European yew, *Taxus baccata*, by Rhone-Poulenc Rorer and Centre National de la Recherche Scientifique, France (Ringel and Horwitz, 1991; Guenard *et al.*, 1993). This agent is an analogue of Taxol (paclitaxel), but has a more hydrophilic chemical structure and requires cremophor as a solvent. The docetaxel formulation was developed with Polysorbate 80 to avoid the use of cremophor, which had been implicated as a possible causative agent in the hypersensitivity reactions (HSRs) seen with paclitaxel (Weiss *et al.*, 1990; Burris *et al.*, 1993). Docetaxel displays a unique mechanism for induction of stable microtubule assembly and promotion of tubulin polymerisation, similar to that of paclitaxel (Ringel and Horwitz, 1991). Docetaxel has been proven to have potent cytotoxic activity in experimental *in vivo* and *in vitro* studies (Bissery *et al.*, 1991, Kelland and Abel, 1992).

The clinical anti-neoplastic efficacy of docetaxel has been studied in phase I and phase II trials in Europe, the United States and Canada (Burris *et al.*, 1993; Piccart, 1993). In breast cancer, high response rates to the drug have been reported by Fumoleau *et al.* (1993), Seidman *et al.* (1993), and Trudeau *et al.* (1993). Similar response rates have been reported for paclitaxel by Holmes *et al.* (1991) and others (Pazdur *et al.*, 1993). Following these pioneering studies, a clinical phase I study of the effects of docetaxel in solid tumours was conducted in Japan (Taguchi *et al.*, 1994*a*). Single and repeated dosing of 1 h intravenous (i.v.) infusions were used, and the maximum tolerated dose (MTD) of docetaxel was 70-90 mg $m^{-2}$, with the dose-limiting factor being leucocytopenia (neutropenia). The recommended dose for further clinical studies in Japan was 60 mg $m^{-2}$. Subsequently, a preliminary phase II study was carried out in Japan to determine the anti-tumour response and safety of docetaxel in breast cancer. Docetaxel 60 mg $m^{-2}$ was given as a 1 h i.v. infusion, for at least two courses every 3-4 weeks (Taguchi *et al.*, 1994*b*). In this study, two complete responses (CRs) and 19 partial responses (PRs) were obtained in 51 eligible patients, and the overall response rate was 41.2%. The present phase II clinical trial was, therefore, designed to confirm the clinical efficacy and tolerability of docetaxel in patients with advanced or recurrent breast cancer. The same dose and schedule was used as in the earlier phase II trial, i.e. 60 mg $m^{-2}$ docetaxel as a 1 h i.v. infusion, given at 3-4 week intervals.

### Patients and methods

### *Eligibility criteria*

Patients were registered from 31 hospitals during the 7 months from March to September 1993 (Registration office, Japanese Society for Cancer Chemotherapy). The majority of patients had recurrent breast cancer; the remaining patients who presented with stage IIIb or IV disease were defined as having advanced disease. Eligibility criteria were as follows: (1) histologically or cytologically confirmed breast cancer with evaluable or measurable lesions; (2) patients could have received adjuvant therapy following mastectomy provided there was a treatment-free period of >6 months since prior adjuvant chemotherapy, or >1 month since prior hormone therapy; (3) patients could have received one or two chemotherapy regimens for advanced or relapsed disease; (4) a wash-out period was required of >4 weeks since prior chemotherapy, or >2 weeks since receiving biological response modifiers, hormones, antimetabolites or radiotherapy (to lesions other than those to be evaluated in the current study); (5) age 15-80 years; (6) JSCT (Japan Society for Cancer Therapy) performance status (PS) of 0-2, which is similar to ECOG PS 0-2; (7) life expectancy more than 3 months after study entry; (8) laboratory parameters of

Correspondence: I Adachi
Received 27 July 1994; revised 7 August 1995; accepted 11 August 1995

leucocyte count 3600–8800 $mm^{-3}$; neutrophil count >2000 $mm^{-3}$; platelet count >100 000 $mm^{-3}$; haemoglobin >9.5 mg $dl^{-1}$; total bilirubin <1.5 mg $dl^{-1}$; serum transaminases (GOT, GPT) <twice the upper limit of normal for the hospital (except for patients with hepatic metastasis); albumin >3.0 g $dl^{-1}$; creatinine within normal range for the hospital.

*Dosage and follow-up observation*

RP56976 (docetaxel), was provided by Rhone-Poulenc Rorer in 2 ml vials as a concentrated solution of 80 mg 2 $ml^{-1}$ in Polysorbate 80. The patients were given at least two cycles of docetaxel 60 mg $m^{-2}$ as a 1 h i.v. infusion. The interval between cycles of treatment was usually 3–4 weeks. However, this was prolonged for up to an additional 2 weeks when incomplete recovery was observed in haematological, blood chemistry and urinary examinations conducted during the course of treatment. Treatment was stopped if grade 4 non-haematological side-effects occurred. Tumour lesions were recorded at least every 3–4 weeks; tumour markers, CEA, CA15-3 and/or others, were examined every 2 or 4 weeks.

*Evaluation of response*

The anti-tumour responses were assessed by extramural review (Appendix 2). Patients with evaluable disease but no measurable lesions were included. We used the JSCT criteria (Furue *et al*., 1986*a*,*b*). These are fundamentally similar to the WHO criteria for evaluating anti-tumour effects and clinical tolerance. However, patients with evaluable but not measurable disease were assessable for response. Improvement of bone metastases was defined as a >50% recalcification or healing of lytic lesions noted on periodical bone X-rays with the aid of the changes in tumour markers and radionuclide scan.

This trial was designed in accordance with the Japanese guidelines for the clinical evaluation of anti-neoplastic drugs (in Japanese; The Ministry of Health and Welfare, 1992), and was performed after the approval of the investigational review board of each hospital was given. A monitoring committee was arranged independently to assess the evaluation of safety and efficacy in the study. Informed consent, usually in writing, was obtained for every patient before entry. Fifty-five patients entered of their own will, but the other 26 patients were registered with the approval of a close relative, i.e. parent, husband, sister, son or daughter. Direct discussion of the diagnosis and/or prognosis with the patient was deemed therapeutically and/or psychologically inappropriate in these cases.

### Results

*Patients*

Of the 81 patients entered in the study, 72 were deemed eligible, and nine were excluded (Table I). The reasons for ineligibility were: no evaluable or measurable tumour lesions ($n=1$), three or more prior chemotherapy regimens ($n=2$), an unacceptably short period since prior therapy ($n=2$), drug treatment with prior registration ($n=3$) and brain metastasis ($n=1$). Treatment efficacy was assessed by intention to treat in all 72 eligible patients, although response could not be determined in three non-evaluable (NE) patients (one due to non-administration of drug, one early death on day 10, and one patient refusal); 71 patients were evaluable for toxicity.

The median age of all 72 patients was 53.5 years (range 29–73 years) and median PS was 0. One patient with PS 4 due to pain from bone metastasis, but who was otherwise well, was included. These 72 patients received a total of 327 cycles of docetaxel, with a median of four cycles each. The dose of docetaxel was modified for six patients on and after the second cycle, five received a reduced dose, approximately 50 mg $m^{-2}$, due to severe neutropenia (<100 $mm^{-3}$) in the prior cycle. The dose was increased to 70 mg $m^{-2}$ in one patient, because of good tolerability during the former cycle.

*Anti-tumour activity*

The anti-tumour response in all 72 eligible patients is shown in Table II. The overall response rate was high at 44.4% (32/72, with 95% CI 32.7–56.6%). The response according to histological tumour type was principally 37.5% for papillotubular carcinoma, 47.8% for solid tubular carcinoma, 40.9% for scirrhous carcinoma and 66.7% for medullary carcinoma.

The correlation between prior therapy and response to docetaxel was examined. The response rate was very high 3/4

**Table I** Patient characteristics

| | |
|---|---|
| Patients entered | 81 |
| Patients eligible | 72 |
| Median age (years) | 53.5 |
| Range | 29–73 |
| Performance status | |
| 0 | 48 |
| 1 | 15 |
| 2 | 8 |
| 4 | 1 |
| Advanced | 9 |
| Recurrent | 63 |
| No prior treatment | 4 |
| Prior treatment | 68 |
| Surgery | 65 |
| Radiotherapy | 19 |
| Chemotherapy | 62 |
| Biological response modifier | 3 |
| Hormonal | 50 |
| Number of lesions (pts) | |
| 1 | 31 |
| 2 | 28 |
| 3 | 7 |
| 4 | 6 |

**Table II** Responses for advanced or recurrent breast cancer and its metastatic sites

| | *Eligible patients* | *CR* | *PR* | *NC(MR)* | *PD* | *NE* | *Response rate (%)* | *Confidence interval (95%)* |
|---|---|---|---|---|---|---|---|---|
| Total response | 72 | 5 | 27 | 24 (5) | 13 | 3 | 44.4 | 32.7–56.6 |
| Advanced | 9 | 1 | 3 | 3 (1) | 1 | 1 | 44.4 | 13.7–78.8 |
| Recurrent | 63 | 4 | 24 | 21 (4) | 12 | 2 | 44.4 | 31.9–57.5 |
| Primary/local | 23 | 6 | 5 | 7 (2) | 4 | 1 | 47.8 | 26.8–69.4 |
| Lymph nodes | 36 | 8 | 15 | 8 (2) | 3 | 2 | 63.9 | 46.2–79.2 |
| Lung | 23 | 2 | 7 | 10 (1) | 3 | 1 | 39.1 | 19.7–61.5 |
| Bone | 21 | | 4 | 12 (1) | 2 | 3 | 19.0 | 5.4–41.9 |
| Liver | 16 | | 8 | 7 (5) | 1 | | 50.0 | 24.7–75.3 |
| Other | 13 | | 1 | 7 (1) | 3 | 2 | 7.7 | 0.2–36.0 |

CR, complete response; PR, partial response; NC, no change; MR, minor response; PD, progressive disease; NE, non-evaluable.

Table III Prior therapy for recurrent patients

| Prior therapy | Eligible patients | CR | PR | NC(MR) | PD | NE | Response rate (%) | Confidence interval (95%) |
|---|---|---|---|---|---|---|---|---|
| No prior treatment | 2 | | 1 | 1 | | | 50.0 | 1.3-98.7 |
| Adjuvant chemotherapy[a] | 40 | 3 | 17 | 13 (3) | 5 | 2 | 50.0 | 33.8-66.2 |
| Including anthracyclines[b] | 17 | | 10 | 5 (1) | 1 | 1 | 58.8 | 32.9-81.6 |
| Non-anthracyclines | 23 | 3 | 7 | 8 (2) | 4 | 1 | 43.5 | 23.2-65.5 |
| Prior chemotherapy[a] | 42 | 2 | 13 | 17(2) | 9 | 1 | 35.7 | 21.6-52.0 |
| Including anthracyclines[b] | 28 | 1 | 8 | 10 (1) | 8 | 1 | 32.1 | 15.9-52.4 |
| Non-anthracyclines | 14 | 1 | 5 | 7 (1) | 1 | | 42.9 | 17.7-71.1 |
| Hormone therapy | 10 | 2 | 6 | 1 (1) | | 1 | 80.0 | 44.4-97.5 |
| Total dose of anthracyclines/prior chemotherapy[c] | | | | | | | | |
| Patients | 26[d] | 1 | 8 | 10 | 6[d] | 1 | | |
| Median dose (mg) | | 180 | 345 | 157.5 | 475 | 600 | | |
| Range (mg) | | | 200-720 | 40-560 | 75-864 | | | |
| Median no. of cycles | | 6 | 5.5 | 3 | 9.5 | 10 | | |
| Range | | | 1-12 | 2-15 | 1-27 | | | |

[a]Includes combination therapy with hormones and chemotherapeutic agents. [b]CA, cyclophosphamide, doxorubicin; CAF, CA + 5-FU; CA-T, CA + tamoxifen citrate. [c]The total volume of anthracyclines was calculated from each volume and total cycles given as prior chemotherpy for recurrent patients. [d]Except two patients treated with unapproved drugs. CR, complete response; PR, partial response; NC, no change; MR, minor response; NE, non-evaluable.

(75%) in patients who had received no previous systemic treatment. Similarly, eight of ten (80%) patients previously treated only with hormone therapy responded to docetaxel (Table III). Among patients previously treated with chemotherapy for recurrent disease, the response rate was similar for those who had received an anthracycline (9/28 = 32%) and those who had not received an anthracycline (6/14 = 43%). The only patient previously treated with two regimens after recurrence showed PD with docetaxel.

The median duration of response at the end of the study was 110 days (range 32-214 days). A decrease in tumour size was often observed in soft tissues (breast or lymph nodes) after the first cycle of treatment but was somewhat delayed in parenchymal tissues (Figure 1). The median time to achieve a response was after the end of the second cycle in the liver and lungs, and after the third cycle in bones. The time to progression is shown in Figure 2. Of the 72 eligible patients, eight were not evaluable for time to progression (three who were NE and five who received other therapy following completion of the study without confirmation of tumour progression). The median time to progression in the remaining 64 patients was 116 days (range 7-239+days). Neither progression of disease nor new lesions were seen in 20 of the 64 patients (31.3%) during or after the study.

**Figure 1** Duration of response for each lesion. Each bar represents median day from initial (left) to terminal (right) of response duration. ▧, Duration of response; ( ): Number of lesions.

**Figure 2** Time to progression in 64 patients. Of an eligible 72 patients, three dropped out as inevaluable and five were excluded from the figure because they were retreated with another therapy before occurrence of progression or newly grown tumour. Median time to progression was 116 days and 20 patients (31.3%) did not have any complication of disease (PD) during the test period.

Table IV Toxicity findings according to JSCT grades[a] ($n$ = 71)

| Event | Grade 1 | 2 | 3 | 4 | Grades 3-4 % |
|---|---|---|---|---|---|
| Decrease of haemoglobin | 35 | 12 | 3 | | 4.2 |
| Leucocytopenia | 2 | 11 | 42 | 14 | 78.9 |
| Neutropenia | 1 | 6 | 7 | 54 | 85.9 |
| Thrombocytopenia | 3 | 1 | 2 | | 2.8 |
| Alopecia | 6 | 29 | 27 | | 38.0 |
| Anorexia | 16 | 18 | 13 | | 18.3 |
| Nausea/vomiting | 21 | 16 | 8 | | 11.3 |
| Fatigue | 22 | 12 | 7 | 0 | 9.9 |
| Fever | 14 | 20 | 1 | | 1.4 |
| Erythema/eruption | 20 | 6 | 1 | 0 | 1.4 |
| Peripheral neuropathy[b] | 16 | 5 | 2 | 0 | 2.8 |
| Diarrhoea | 13 | 5 | 1 | 0 | 1.4 |
| Oedema[c] | 6 | 7 | 2 | 0 | 2.8 |
| Stomatitis | 8 | 4 | 2 | 0 | 2.8 |
| Pain (arthralgic, muscular, bone) | 7 | 4 | 1 | 0 | 1.4 |
| Allergy | 4 | 1 | 0 | 0 | 0.0 |
| Pruritus | 2 | 1 | 0 | 0 | 0.0 |
| Skin abrasion | 2 | 0 | 0 | | 0.0 |
| Renal failure | 0 | 0 | 0 | 1 | 1.4 |
| Phlebitis | 0 | 0 | 0 | 1 | 1.4 |
| Palpitation | 1 | 0 | 0 | 0 | 0.0 |
| Other[d] | 10 | 3 | 1 | 0 | 1.4 |

[a]Recorded according to the Japan Society for Cancer Therapy toxicity classification criteria (Furue et al., 1986b). [b]Includes hypesthesia, sensory or taste disorder and sensation of heat. [c]One patient showed pleural effusion (grade 2) accompanied by oedema (grade 1). [d]Includes headache ($n$ = 5: grade 1), abdominal pain ($n$ = 2: grade 1, $n$ = 2: grade 2), vertigo ($n$ = 1: grade 1), pigmentation ($n$ = 1: grade 1), tremor ($n$ = 1: grade 1), conjunctivitis ($n$ = 1: grade 2) and constipation ($n$ = 1: grade 3).

### *Toxicity*

Toxicity is shown in Table IV. Leucocytopenia and neutropenia were the major toxicities of docetaxel, with grade 3/4 toxicity occurring in 78.9% and 85.9% respectively of the 71 patients. In 56 patients who had an episode of leucocytopenia without granulocyte colony-stimulating factor (G-CSF) treatment, leucocytopenia or neutropenia was brief and reversible; the median timing of nadir was at days 8–8.5 leucocyte count and days 9–10 (neutrophil) in cycles 1–3 (Figure 3). However, there was no increase in severity in subsequent cycles, and the leucocyte count recovered 20–24 days after treatment. Thrombocytopenia and anaemia were seen less frequently. Febrile episodes occurred in 35 patients, (49.3%), ⩾38°C fever accompanying neutropenia occurred in only eight patients (11.3%).

Alopecia also occurred frequently, being seen in 62/71 patients (87.3%), and was complete (grade 3) in 27 patients (38%). The incidence of grade 3 anorexia, nausea/vomiting, and fatigue was in each case less than 20%. Acute HSR, i.e. face flushing and palpitation, were observed in cycles 1–3 in one patient, who received a total of six cycles of docetaxel. Such episodes which were not very severe, were made tolerable by corticosteroid (hydrocortisone) medication in the second and third cycles, and disappeared with premedication of dexamethasone, diphenhydramine and ranitidine hydrochloride in the subsequent 4–6 cycles.

Oedema occurred in 15 patients (21.1%), and a severe, grade 3 episode was recorded in one patient each in the earlier cycles (cycles 1, 3 and 4). Oedema was first documented after a median of four cycles or 240 mg m$^{-2}$

Each value of leucocytopenia (neutropenia)

| Cycle | No. of patients | Median nadir | Median days of nadir | Median days from nadir to recovery | Median days in grade 3–4 |
|---|---|---|---|---|---|
| First | 56(56) | 1450(358) | 8.5(9) | 8.5(9) | 2(4) |
| Second | 51(43) | 1600(300) | 8(10) | 12(10.5) | 1.5(4) |
| Third | 37(34) | 1600(387) | 8(10) | 14.5(13) | 3(3) |

**Figure 3** Leucocytopenia and neutropenia of patients without G-CSF. Leucocyte count is demonstrated (top) showing first cycle of 56 patients (-○-), second cycle of 51 patients (-□-) and third of 37 patients (-△-) respectively. Neutrophils were also plotted bottom, as first cycle of 56 patients (-●-), second cycle of 47 patients (-■-) and third of 34 patients (-▲-) respectively. Leucocytopenia was severe but immediately reversible in the first cycle, and found to be more or less extended in the second and third cycles. However, the data of median days of nadir and those in grade 3 or 4 leucocytopenia and/or neutropenia demonstrated that they did not cumulatively increase in severity in any period. Neutropenia followed leucocytopenia as shown by median days of nadir. Each value and error bar represents mean and s.e.

of docetaxel. No prophylactic medication was given to prevent oedema. However, 13 (86.7%) patients received diuretics (frusemide or spironolactone) therapeutically after the occurrence of oedema. Skin toxicity involving erythema/eruptions and/or skin abrasions occurred, but nail changes or other severe skin toxicity findings were not observed throughout the study. In terms of cardiac toxicity, mild palpitations were observed in one patient. Grade 4 phlebitis at the injection site was observed in one patient, and was thought to have arisen from drug leakage.

A patient, 67 years of age, who had widespread local and metastatic disease affecting both lungs, lymph nodes and bone, died 10 days after her first cycle of 60 mg m$^{-2}$ (total 78 mg) docetaxel. Clinical signs of diarrhoea and fever of more than 37°C were noted immediately after dosing. Stomatitis and severe grade 4 neutropenia subsequently occurred on days 6 and 7 respectively. Acute renal failure, characterised by increases in blood urea nitrogen (BUN) and creatinine values, was detected 9 days before the occurrence of dyspnoea, but recovered temporarily with diuretic treatment. Death was attributed to pulmonary congestion accompanying grade 4 acute renal failure.

### Discussion

The efficacy of docetaxel, 60 mg m$^{-2}$ dose, in breast cancer has previously been demonstrated. A response rate of 42% was reported in patients who had not responded to standard chemotherapy in an early phase II clinical trial in Japan (Taguchi *et al*., 1994*b*). Subsequently, two independent multi centre late phase II studies, consisting of two different groups (A and B) have been conducted. These studies were the same in design and evaluated by the same review board determining patient eligibility and anti-tumour response. The present study (group B) with a response rate of 44% confirmed that 60 mg m$^{-2}$ of docetaxel was effective in patients with advanced or recurrent breast cancer. In another late phase II trial (group A) a similar high response rate of 52.2% has been reported (Taguchi *et al*., 1994*c*).

Seidman *et al*. (1993) reported a high potency, i.e. a response rate of 57% (8/14), in patients with metastatic breast cancer. This phase II study in the USA used docetaxel 100 mg m$^{-2}$ administered as a 1 h i.v. infusion as first-line

First documentation of peripheral oedema

| Cycle | 1 | 2 | 3 | 4 | 5 | 6 | 7 | 8 | 9 | 10 |
|---|---|---|---|---|---|---|---|---|---|---|
| Patients | 2 | 1 | 3 | 6 | 1 | 2 | 0 | 0 | 0 | 0 |

**Figure 4** Occurrence of peripheral oedema. Peripheral oedema occurred in 15 patients. Oedema was not intensified in severity by repeated dosage, while the incidence (%) increased as a result of fewer patients in later cycles. Columns represent total patients (□), and patients with grade 1–2 (▨) or grade 3 (■) oedema.

chemotherapy. A similar response rate, 57% (12/21), was achieved with the same dose in Canada (Trudeau et al., 1993). In EORTC phase II studies of patients with advanced breast cancer, Fumoleau et al. (1993) reported a higher response rate of 73% (24/33) with the 100 mg $m^{-2}$ dose. By contrast, Dieras et al. (1994) recently reported a response rate of 50.0% with 75 mg $m^{-2}$ docetaxel given without any premedication. Therefore, anti-tumour activity of this agent probably depends on the dose, a higher dose close to the MTD clinically more effective. However, in the Japanese clinical trials, a lower dose of 60 mg $m^{-2}$ was recommended. Doses of 70 mg $m^{-2}$ or higher were rejected as this was the MTD in our phase I study (Taguchi et al., 1994a). Generally in Japanese patients, the combination of cyclophosphamide (CPA)–doxorubicin (DOX) with or without 5FU or CPA–methotrexate–5FU, is used as first-line chemotherapy. These regimens have been demonstrated as highly effective against metastatic and recurrent breast cancer (Kanda et al., 1981; Kubo et al., 1983). However, the duration of response is relatively short, 8–12 months, and there are few if any anti-neoplastic drugs active as second- or third-line chemotherapy against DOX-resistant cancer. The present study demonstrated that the response rate in patients who had received prior DOX chemotherapy was little different from that in patients not exposed to anthracyclines. This relatively high potency is notable in comparison with other drugs used in patients after prior DOX therapy, strongly indicating that docetaxel is a candidate for second-line chemotherapy of DOX-resistant breast cancer.

The median duration of response and median time to progression, each approximately 3 months, were similar to those reported in the EORTC phase II study (Fumoleau et al., 1993). We believe that the results of the current study are better than those achieved with standard chemotherapy, since docetaxel was used as second-line treatment in the majority of patients (94.4%). Gregory et al. (1993) demonstrated in their extensive study of 1756 breast cancer patients that the median duration of response was 7.8 months and the median time to progression was 3.7 months with first-line chemotherapy. However, after two or more chemotherapy regimens these periods were significantly shortened to 2.3 months. Further studies will be required to evaluate the impact on survival and/or quality of life of docetaxel as first-line chemotherapy in recurrent breast cancer.

The dose-limiting toxicity of docetaxel has already been demonstrated to be leucocytopenia or neutropenia in phase I and early phase II studies in Japan (Taguchi et al., 1994a,b). Similarly, episodes of neutropenia were found to be severe but reversible and of brief duration in the present study. Additionally, there was a small proportion of patients who developed febrile neutropenia on treatment with a dose of 60 mg $m^{-2}$. G-CSF was administered to them and the recovery time was shortened, despite the severity of the neutropenia.

In the present study, we also found mild HSR effects, such as skin rash and pruritus, and one patient had acute HSR. HSR has been reported in a phase I study of docetaxel, and Burris et al. (1993) proposed that HSR might be due to the basic 'taxane' molecule itself. In the EORTC study, Wanders et al. (1993) reported HSR following docetaxel in 27% of the 337 patients. However it recovered upon discontinuation of the infusion and appropriate therapy with corticosteroids, anti-histamines and $H_2$-antagonists. A patient having acute HSR in the present study was able to continue receiving the docetaxel when the dosing interval was prolonged or with the administration of steroid. Moreover, HSR was also prevented by prophylactic medication with steroids and $H_1$- and $H_2$-antagonists in the following cycles. Thus, we believed that HSR was mild and tolerable at a dose of 60 mg $m^{-2}$ of docetaxel.

The incidence of oedema in this study was 21.1%. Oedema has been reported to be cumulative, since it has been detected after several (5–6) cycles in previous studies (Fumoleau et al., 1993; Piccart 1993). Oedema after treatment with docetaxel has been divided into two distinct types. Firstly, angioedemea, which is responsive to corticosteroids; secondly a fluid retention syndrome, manifesting as peripheral oedema or pleural effusion that is responsive to diuretics (Pazdur et al., 1993). In the present study episodes of oedema seemed to be of the latter type, i.e. peripheral oedema, which was tolerable and reversible on diuretic treatment. Oedema appears to have occurred after fewer cycles and a lower docetaxel dose than reported using the 100 mg $m^{-2}$ dose. The development of oedema probably depends on the number of cycles rather than the total amount of the agent. The reason for the apparently earlier occurrence of oedema may be that few patients received a large number of cycles of docetaxel. Oedema occurred repeatedly in a few patients, but did not increase in severity. Therefore, the patients who had peripheral oedema were able to continue treatment with docetaxel.

The therapy-related death in this study was considered to be a result of severe dyspnoea accompanying acute renal failure. The precise relationship between the death and the acute renal failure is not yet known. However, dyspnoea has been suggested to be related to an increase of pleural fluid arising from pleurisy with bilateral pulmonary metastasis and drug-related oedema.

The recent EORTC phase II study recommended a docetaxel dose of 100 mg $m^{-2}$ since the activity is greater than, but the safety profile similar to, that for a dose of 75 mg $m^{-2}$ (Dieras et al., 1994). However, in the current study toxicity was mild using lower doses of docetaxel. Neutropenia was marked but reversible; there was a low incidence of HSR and/or oedema, which were tolerable without any prophylactic medication. Moreover, ethically and clinically we could not use higher doses of docetaxel given the results of phase I trials in Japan. In conclusion, we found a dose of 60 mg $m^{-2}$ of docetaxel without premedication to be sufficient in terms of clinical activity and tolerability for advanced or recurrent breast cancer.

**Acknowledgements**
We are grateful to the many doctors who participated in RP56976 clinical study group B for breast cancer (see Appendix 1). We also thank Drs S Tsukagoshi, M Yoshida, I Nakao and Y Ohashi, who are members of the monitoring committee, for their helpful suggestion in evaluating the safety and effectiveness of the study. This study was supported in part by Grants-in-Aid for Cancer Research (2S-1, 5-28), second-term Comprehensive 10 year Strategy for Cancer Control and by funds from Chugai Pharmaceutical and Rhone-Poulenc Rorer.

Appendix 1 RP56976 Clinical study group B for breast cancer

| | |
|---|---|
| Koichi Hirata, Minoru Okazaki | 1st Dept. of Surgery, Sapporo Medical College |
| Masami Ogita | Dept. of Surgery, Sapporo National Hospital |
| Yuh Sakata, Hidekazu Suzuki | Dept. of Internal Medicine, Aomori Prefectural Central Hospital |
| Yasuo Kunii, Naonori Takahashi | Dept. of Surgery, Sendai National Hospital |
| Takahiro Satomi | Dept. of Surgery, Iwaki Kyoritu General Hospital |
| Morihiko Kimura | Dept. of Surgery, Gunma Cancer Center, Tohmo Hospital |
| Jiro Ando | Dept. of Surgery, Tochigi Cancer Center |
| Toshikazu Suwa, Tsuyoshi Saito | Dept. of Surgery, Omiya Red Cross Hospital |
| Naokazu Nagata, Yoko Nemoto, Yasuyuki Katayama | 3rd Dept. of Internal Medicine, Dept. of Laboratory Medicine, National Defense Medical College |
| Yoshiya Mishina | Dept. of Breast Surgery, Kameda General Hospital |

| | |
|---|---|
| Muneaki Sano, Haruhiko Makino | Dept. of Surgery, Niigata Cancer Center Hospital |
| Isamu Adachi, Toru Watanabe, Masaru Narabayashi, Rumiko Okamoto | Dept. of Medical Oncology, National Cancer Center Hospital |
| Noboru Horikoshi, Toshiki Uchida, Hiroyoshi Mihara, Ryo Sobue | Dept. of Medical Oncology, Center Institute Hospital, Japanese Foundation for Cancer Research |
| Harubumi Kato, Tetsuya Okunaka, Haruya Koshi-ishi | 1st Dept. of Surgery, Tokyo Medical College |
| Yukihide Isogai, Yasunobu Kuraishi, Tadashi Kobayashi, Tadashi Nakamura | 3rd Dept. of Internal Medicine, The Jikei University School of Medicine |
| Tsunehiro Nishi, Atsushi Fukuuchi | Dept. of Breast & Endocrine Surgery, Mitsui Memorial Hospital |
| Shigeto Miura, Toru Takeuchi | Dept. of Breast Surgery, Aichi Cancer Center |
| Hideaki Aoyama | Dept. of Surgery, National Nagoya Hospital |
| Kaoru Miura, Katsumi Iwase | Dept. of Surgery, Fujita Health University School of Medicine |
| Itsuo Miyazaki, Kazuo Kinoshita, Naomi Nojima, Gen Arakawa | 2nd Dept. of Surgery, School of Medicine, Kanazawa University |
| Masayoshi Mai, Yasushi Deguchi | Dept. of Surgery, Cancer Research Institute, Kanazawa University |
| Masayuki Imamura, Norimichi Kan | 1st Dept. of Surgery, Kyoto University, Faculty of Medicine |
| Hiroaki Kinosita, Ken Morimoto | 2nd Dept. of Surgery, Osaka City University Medical School |
| Yuichi Takatsuka, Eisei Shin | Dept. of Surgery, Osaka National Hospital |
| Hiroki Koyama, Kazuyoshi Motomura | Dept. of Surgery, The Center for Adult Disease, Osaka |
| Masaru Miyashita, Maki Kubota | Dept. of Surgery, Kohnan Hospital |
| Kunzo Orita, Fumiyuki Inoue, Yasunori Ishii | 1st Dept. of Surgery, Okayama University, Medical School |
| Shigemitsu Takashima, Akira Kurita, Ryuichiro Ohashi, Kazuhito Minami | Dept. of Surgery, National Shikoku Cancer Center |
| Masao Tanaka, Syoji Kuroki | Dept. of Surgery 1, Kyusyu University, Faculty of Medicine |
| Takayuki Shirakusa, Teru Hideshima | 2nd Dept. of Surgery, School of Medicine, Fukuoka University |
| Kazuo Tamura, Emi Ishikawa | Dept. of Internal Medicine, Miyazaki Prefectural Hospital |

Appendix 2 Extramural review board

| | |
|---|---|
| Yasutsuna Sasaki | Division of Hematology/Oncology, National Cancer Center Hospital East |
| Takashi Fukutomi | Department of Surgery, National Cancer Center Hospital |
| Koji Enomoto | Department of Surgery, School of Medicine, Keio University |
| Ken Morimoto | 2nd Department of Surgery, Osaka City University Medical School |
| Yuichi Takatsuka | Department of Surgery, Osaka National Hosiptal |
| Hiroki Koyama | Department of Surgery, The Center for Adult Disease, Osaka |
| Jun Ota | Department of Surgery, Hanwa Sumiyoshi General Hospital |
| Tomio Wada | 1st Department of Surgery, Kinki University School of Medicine |
| Hiroshi Sonoo | Department of Endocrincology/Surgery, Kawasaki Medical School |
| Shigemitsu Takashima | Department of Surgery, National Shikoku Cancer Center |

### References

BISSERY M-C, GUENARD D, GUERITTE-VOEGELEIN F AND LAVELLE F. (1991). Experimental antitumour activity of taxotere(RP56976, NSC628503), a taxol analogue. *Cancer Res.*, **51,** 4845–4852.

BURRIS H, IRVIN R, KUHN J, KALTER S, SMITH L, SHAFFER D, FIELDS S, WEISS G, ECKARDT J, RODRIGUEZ G, RINALDI D, WALL J, COOK G, SMITH S, VREELAND F, BAYSSAS M, LEBAIL N AND VON HOFF D. (1993). Phase I clinical trial of taxotere administered as either a 2-hour or 6-hour intravenous infusion. *J. Clin. Oncol.*, **11,** 950–958.

DIERAS V, FUMOLEAU P, CHEVALLIER B, KERBRAT P, KRAKOWSKY Y, ROCHE H, MISSET JL, LENTZ MA, AZLI N AND POUILLART P. (1994). Second EORTC-clinical screening group(CSG) phase II trial of Taxotere (docetaxel) as first line chemotherapy in advanced breast cancer (ABC). *Proc. Am. Soc. Clin. Oncol.*, **13,** 78(115).

FUMOLEAU P, CHEVALLIER B, KERBRAT P, DIERAS V, LE BAIL N, BAYSSAS M AND VAN GLABBEKE M. (1993). First line chemotherapy with taxotere(T) in advanced breast cancer(ABC): a phase II study of the EORTC clinical screening group(CSG). *Proc. Am. Soc. Clin. Oncol.*, **12,** 56(27).

FURUE H, HARA Y, IMAI Y, KIMURA T, KOYAMA Y, KURIHARA M, MAJIMA H, NAKAO I, NIITANI H, OGAWA M, ONOSHI T, SAITO T, SAKAI Y, SAKANO T, SAKUMA A, TAKAYA O, TOMINAGA S AND YOKOYAMA M., eds. (1986*a*). Criteria for the evaluation of direct effects of solid cancer chemotherapy (in Japanese) *J. Jpn. Soc. Cancer Ther.*, **21,** 931–942. (English translated version: *J. Jpn. Soc. Cancer Ther.*, **28,** 105–118, 1993).

FURUE H, HARA Y, IMAI Y, KIMURA T, KOYAMA Y, KURIHARA M, MAJIMA H, NAKAO I, NIITANI H, OGAWA M, ONOSHI T, SAITO T, SAKAI Y, SAKANO T, SAKUMA A, TAKAYA O, TOMINAGA S AND YOKOYAMA M., eds. (1986*b*). Criteria for the evaluation of effect reinforcement of solid cancer chemotherapy (in Japanese) *J. Jpn. Soc. Cancer Ther.*, **21,** 943–953. (English translated version: *J. Jpn. Soc. Cancer Ther.*, **28,** 119–130, 1993).

GREGORY WM, SMITH P, RICHARDS MA, TWELVES CJ, KNIGHT RK AND RUBENS RD. (1993). Chemotherapy of advanced breast cancer: outcome and prognostic factors. *Br. J. Cancer*, **68,** 988–995.

GUENARD D, GUERITTE-VOEGELEIN F. AND POTIER P. (1993). Taxol and taxotere: discovery, chemistry, and structure-activity relationships. *Acc. Chem. Res.*, **26,** 160–167.

HOLMES FA, WALTERS RS, THERIAULT RL, FORMAN AD, NEWTON LK, RABER MN, BUZDAR AU, FRYE DK AND HORTOBAGYI GN. (1991). Phase II trial of taxol, an active drug in the treatment of metastatic breast cancer. *J. Natl Cancer Inst.*, **83,** 1797–1805.

KANDA K, YAMAGATA J, TAKENAKA K, TASHIRO H AND NOMURA Y. (1981). Combination chemotherapy of metastatic breast cancer with 5-fluorouracil, adriamycin, cyclophosphamide (FAC). *Jpn. J. Cancer Chemother.*, **8,** 749–756.

KELLAND LR AND ABEL G. (1992). Comparative *in vitro* cytotoxicity of taxol and taxotere against cisplatin-sensitive and -resistant human ovarian carcinoma cell lines. *Cancer Chemother. Pharmacol.*, **30,** 444–450.

KUBO K, ABE O, IZUO M, ENOMOTO K, KOYAMA H, SAKAI K, TERASAWA T, TOMINAGA T AND NOMURA Y. (1983). Clinical evaluation of adriamycin for advanced breast cancer. III. A joint study by 26 institutes on the 'CAF' and 'CMcF' regimens. *Jpn. J. Cancer Chemother.*, **10,** 2523–2531.

PAZDUR R, KUDELKA AP, KAVANAGH JJ, COHEN PR AND RABER MN. (1993). The taxoids: paclitaxel (Taxol) and docetaxel (Taxotere). *Cancer Treat. Rev.*, **19,** 351–386.

PICCART MJ. (1993). Taxotere: a second generation taxoid compound. In *ASCO Educational Book*, Dalton, WS *et al.* eds, pp. 25–32. American Society of Clinical Oncology.

RINGEL I AND HORWITZ SB. (1991). Studies with RP56976 (taxotere): a semisynthetic analogue of taxol. *J. Natl Cancer Inst.*, **83,** 288–291.

SEIDMAN AD, HUDIS C, CROWN JPA, BALMACEDA C, LEBWOHL D, CURRIE V, GILEWSKI T, HAKES T, ROBLES M, KLEM K, LEPORE J AND NORTON L. (1993). Phase II evaluation of taxotere (RP56976, NSC628503) as initial chemotherapy for metastatic breast cancer. *Proc. Am. Soc. Clin. Oncol.*, **12**, 63(52).

TAGUCHI T, FURUE H, NIITANI H, ISHITANI K, KANAMARU R, HASEGAWA K, ARIYOSHI H, NODA K, FURUSE K, FUKUOKA M, YAKUSHIJI M AND KASHIMURA M. (1994*a*). Phase I clinical trial of RP56976 (docetaxel), a new anticancer drug (in Japanese, abstract in English). *Jpn. J. Cancer Chemother.*, **21**, 1997–2005.

TAGUCHI T, HIRATA K, KUNII Y, TABEI T, SUWA T, KITAJIMA M, ADACHI I, TOMINAGA T, SHIMADA H, SANO M, MIURA S, TAKAI S, KINOSHITA H, MATSUNAGA S, KONISHI Y, SONOO H, HIRAKI T, TOGE T, TAKASHIMA S, TANAKA M, OGAWA M AND OTA J. (1994*b*). An early phase II clinical study of RP56976 (docetaxel) in patients with breast cancer (in Japanese, abstract in English). *Jpn. J. Cancer Chemother.*, **21**, 2453–2460.

TAGUCHI T, MORI S, ABE R, HASEGAWA K, MORISHITA Y, TABEI T, SASAKI Y, FUJITA M, ENOMOTO K, HAMANO K, TOMINAGA T, SASAKI T, YAMAGUCHI S, NISHIYAMA K, IIDA F, KANDA K, TAKAGI H, MASAOKA A, TAKAHASHI T, OKA T, TAKAI S, OTA J, WADA T, YAYOI K, NAITO Y, KONISHI Y, SONOO H, HIRAKI S, YAMAMOTO Y, NAKASE A, DOHI K, MONDEN Y AND OGAWA M. (1994*c*). Late phase II clinical study of RP56976 (docetaxel) in patients with advanced/recurrent breast cancer (in Japanese, abstract in English). *Jpn. J. Cancer Chemother.*, **21**, 2625–2632.

TRUDEAU ME, EISENHAUER E, LOFTERS W, NORRIS B, MULDAL A, LETENDRE F, VANDENBURG T AND VREMA S. (1993). Phase II study of taxotere as first line chemotherapy for metastatic breast cancer (MBC). A national cancer institute of Canada clinical trials group (NCIC CTG) study. *Proc. Am. Soc. Clin. Oncol.*, **12**, 64 (59).

WANDERS J, SCHRIJVERS D, BRUNTSCH U, GORE M, VERWEIJ J, HANAUSKE AR, FRANKLIN H, ROELVINK M, BAYSSAS M AND KAYE SB. (1993). The EORTC–ECTG experience with acute hypersensitivity reactions (HSR) in taxotere studies. *Proc. Am. Soc. Clin. Oncol.*, **12**, 73 (94).

WEISS RB, DONEHOWER RC, WIERNIK PH, OHNUMA T, GRELLA RJ, TRUMP DL, BAKER JR Jr, VANECHO DA, VONHOFF DD AND LEYLAND-JONES B. (1990). Hypersensitivity reactions from taxol. *J. Clin. Oncol.*, **8**, 1263–1268.